# System Development Guidelines From a Review of Motion-Based Technology for People With Dementia or MCI

**DOI:** 10.3389/fpsyt.2018.00189

**Published:** 2018-05-11

**Authors:** Arlene J. Astell, Stephen Czarnuch, Erica Dove

**Affiliations:** ^1^Research and Academics, Ontario Shores Centre for Mental Health Sciences, Whitby, ON, Canada; ^2^Occupational Sciences and Occupational Therapy, University of Toronto, Toronto, ON, Canada; ^3^School of Psychology and Clinical Language Sciences, University of Reading, Reading, United Kingdom; ^4^Department of Electrical and Computer Engineering/Discipline of Emergency Medicine, Memorial University of Newfoundland St. John's, NL, Canada

**Keywords:** dementia, mild cognitive impairment, motion-based technology, system design, design guidelines

## Abstract

As the population ages and the number of people living with dementia or mild cognitive impairment (MCI) continues to increase, it is critical to identify creative and innovative ways to support and improve their quality of life. Motion-based technology has shown significant potential for people living with dementia or MCI by providing opportunities for cognitive stimulation, physical activity and participation in meaningful leisure activities, while simultaneously functioning as a useful tool for research and development of interventions. However, many of the current systems created using motion-based technology have not been designed specifically for people with dementia or MCI. Additionally, the usability and accessibility of these systems for these populations has not been thoroughly considered. This paper presents a set of system development guidelines derived from a review of the state of the art of motion-based technologies for people with dementia or MCI. These guidelines highlight three overarching domains of consideration for systems targeting people with dementia or MCI: (i) cognitive, (ii) physical, and (iii) social. We present the guidelines in terms of relevant design and use considerations within these domains and the emergent design themes within each domain. Our hope is that these guidelines will aid in designing motion-based software to meet the needs of people with dementia or MCI such that the potential of these technologies can be realized.

## Introduction

Dementia is a progressive neurodegenerative condition of multiple causes (e.g., Alzheimer's disease, vascular disease) which produces noticeable impairments in areas of cognitive functioning such as memory, attention, communication, comprehension and executive function (1, 2). Mild cognitive impairment (MCI) is defined as a condition in which a person shows mild yet measurable cognitive changes greater than those expected for their age; however, these changes do not impair the person's ability to perform activities of daily living, such as those experienced by people with dementia (3). MCI increases the risk of a person developing dementia (3) although this is not inevitable. As the population continues to age and life expectancy continues to increase (4), the prevalence of conditions such as dementia and MCI is also growing (5). For instance, the number of people living with dementia globally is expected to increase three-fold to 131.5 million by 2050 (5). In the absence of pharmacological or other treatments to reverse these conditions, it is increasingly important to identify interventions that can support people with dementia and MCI to live well (6, 7).

“Exergaming” (combining exercise and recreation) using Nintendo Wii and Xbox Kinect is growing in popularity for people with dementia or MCI (henceforth, people with cognitive impairment: PCI), and also as a tool for scientific research (7–10). Motion-based technology is an immersive and intuitive type of technology that relies purely on natural gestures and physical motions for interaction (e.g., waving an arm). The current literature highlights the vast potential of these technologies to improve the lives of PCI through participation in cognitive, physical and leisure activities (7). This is relevant to developers of technological interventions and software targeting PCI in broad areas such as game development [e.g., (11)], automated assessments [e.g., (12)], and ambient and smart environments [e.g., (13)]. However, there is a significant gap in information regarding optimal ways for motion-based technology to be introduced, taught, supported and used with PCI. For example, a comprehensive systematic review of motion-based technology interventions involving PCI found only 31 unique studies (7). Of those studies, only 19 implemented procedures for promoting use and competence with the motion-based technology. While these exploratory studies shed light on the importance of research in this area, the level of detail provided regarding the introduction, teaching and support methods applied when using motion-based technologies with PCI varied significantly. Furthermore, many of these studies featured small sample sizes, limiting the generalizability of the findings. Nevertheless, in the absence of a strong body of literature to draw on, (7) justifiably recommended that the methods implemented in the 19 studies be thematically categorized into initial design guidelines, and incorporated directly into future research and development with PCI using motion-based technologies to further increase the potential of this type of technology (7).

In response to this recommendation, we present a set of system development guidelines derived from the current literature on motion-based technologies with application to PCI. The aim of these guidelines is to foster optimal development of motion-based technology systems and interventions for PCI, ultimately increasing the usability and efficacy of these technologies and the systems and researchers that use them. Specifically, these guidelines are targeted at developers of technology and researchers who seek to effectively collect data directly from motion-based technology, or develop interventions using these technologies, while simultaneously promoting fun and meaningful engagement for PCI. Our recommendations are timely, given that developing guidelines for technologies being used with PCI is becoming more prevalent. For example, Ben-Sadoun et al. (14) recently created design recommendations for the development of serious games being used with PCI.

## Relevant literature

In recent years, there have been an increasing number of research interventions involving motion-based technology systems and PCI (7). To capture a complete, exhaustive summary of this growing yet uncharted body of literature, Dove and Astell (7) completed a systematic review, finding that motion-based technology can be used to provide cognitive, physical, and leisure activities to PCI, and that these activities are perceived as engaging and enjoyable by these populations (7). These findings highlight the broad potential of motion-based technology as an effective yet engaging intervention or data collection tool for use with PCI. However, to further increase the potential of this type of technology for PCI, one key recommendation emphasized by the authors was the need for design guidelines that could be directly integrated into future research and development of activities or interventions featuring motion-based technology (7), such as gaming, automated assessment and intelligent environments. While design guidelines have been created for people with Alzheimer's disease or related dementias, these guidelines have not focused on motion-based technologies specifically, and have not been expanded beyond the use of games (14–17). Additionally, there are guidelines related to motion-based technologies geared toward healthy older adults or other rehabilitative populations with physical impairments (18–23) but none are specific to people living with cognitive impairments. As such, there is still an existing need to produce broad guidelines related to motion-based systems designed for PCI. Accordingly, the main objective of this research is to propose design considerations for developers and researchers who use motion-based systems with PCI based on a review of the current research related to motion-based system use with these populations.

## Methods

**Figure 1:**
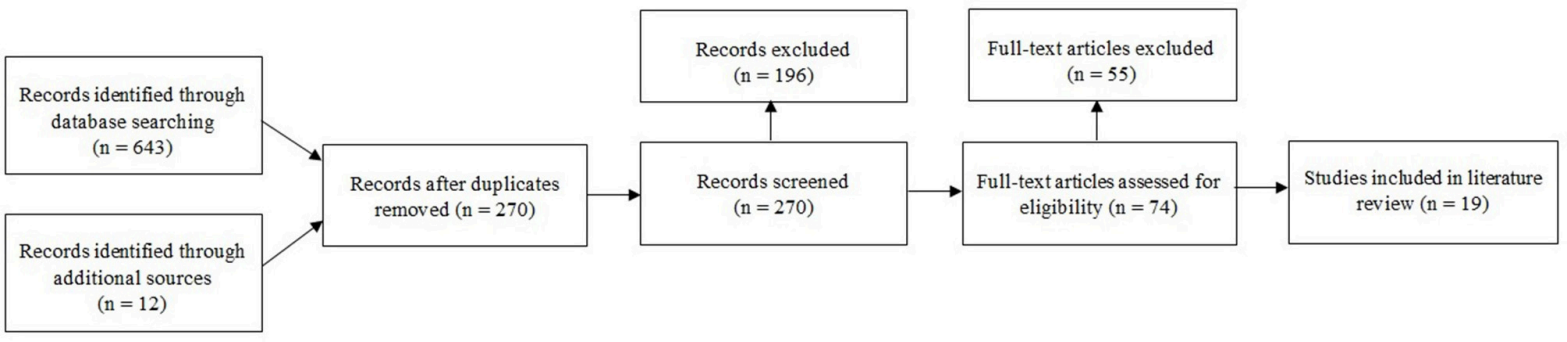
Flow diagram of search procedure.

We searched the PubMed (NCBI), CINAHL (EBSCO Health), PsycINFO (Ovid), MEDLINE (Ovid), and Cochrane Database of Systematic Reviews (Ovid) electronic databases. All database results were restricted to 2006–2016 to reflect the maturity of motion-based technologies in research. To focus on the use of motion-based technology systems for use with PCI, we developed three search word strings according to population, technology and application. Our population search terms were: “dementia”, “Alzheimer^*^,” “mild cognitive impairment,” and “MCI.” Our technology search terms were: “exergam^*^,” “motion-based,” “virtual reality,” “gesture-based.” “Nintendo Wii,” “interactive console,” and “Xbox Kinect.” Our application search terms were: “activit^*^” and “gam^*^.” In all the above search terms, the “^*^” symbol represents a wildcard character to allow for variable endings of a root word. To be included in the review, resulting research needed to meet the following inclusion criteria: written in English; reporting a study with PCI; involving a motion-based technological intervention; and including mention of introducing, teaching or supporting participants to use the technology. A total of 643 articles, book chapters and conference papers were identified through the initial database queries. Duplicate documents resulting from listings in multiple databases were removed, yielding 270 articles that were evaluated against the inclusion criteria. A total of 19 articles met the inclusion criteria, were fully reviewed and were included in the study as the research data (see Figure 1 for a description of the search procedure).

The data were collated, charted, fully read and summarized according to the recommendation of Dove and Astell (7) (see Table 1). We utilized a synthesis approach to analyze the data (24), and design considerations were iteratively coded. The design considerations, in the context of motion-based system development and use, were grouped into similar patterns, forming overarching domains of design consideration. Finally, we explored within each domain, searching for similarities across the included studies, allowing themes to emerge. The result was a set of domains with specific themes in each domain highlighting design considerations relevant to PCI.

**Table 1:** Summary of articles identified for inclusion.

**References**	**Participants (*n*)**	**Study population**	**Purpose of MBT use**
(25)	*n =* 322	Dementia and MCI	Cognitive function
(26)	*n =* 9	Dementia	Leisure activities
(27)	*n =* 14	Dementia and MCI	Physical function/activity promotion
(28)	*n =* 2	Dementia	Cognitive function
(29)	*n =* 29	Dementia	Leisure activities
(30)	*n =* 13	Dementia	Cognitive function, physical function/activity promotion, and leisure activities
(31)	*n =* 3	Dementia	Cognitive function
(32)	*n =* 50	MCI	Cognitive function
(33)	*n =* 53	Dementia and MCI	Physical function/activity promotion and leisure activities
(34)	*n =* 20	MCI	Cognitive function
(35)	*n =* 116	Dementia	Physical function/activity promotion
(36)	*n =* 1	Dementia	Cognitive function and leisure activities
(37)	Unspecified	Dementia and MCI	Cognitive function
(38)	*n =* 1	Dementia	Physical function/activity promotion
(39)	*n =* 20	Dementia	Leisure activities
(40)	*n =* 10	Dementia	Physical function/activity promotion and leisure activities
(41)	*n =* 22	Dementia	Cognitive function, physical function/activity promotion and leisure activities
(42)	*n =* 79	Dementia and MCI	Cognitive function and leisure activities
(43)	*n =* 2	MCI	Cognitive function and leisure activities

## Motion-based technology system development guidelines

Our analysis resulted in three overarching domains of design consideration: (i) cognitive, (ii) physical and (iii) social. Within the cognitive, physical and social domains, seven, six and three themes emerged respectively. We now present the findings of each major design theme, organized by domain (see Table 2 for a descriptive overview).

**Table 2:** Summary of motion-based technology design features for people with dementia and MCI.

**Cognitive**	**Physical**	**Social**
Clear goal	Accommodate mobility aids	Tailor to interests
Maximize retained skills	Account for imprecise motor control	Design for an audience
Understandable, appropriate instructions	Age-appropriate physical component	Positive, timely feedback
Effective use of prompts	Intuitive user interaction	
Avoid timed responses	Adaptable to physical variances	
Gain and sustain attention	Audio-visual accommodations	
Failure-free		

### Cognitive domain considerations

The cognitive capabilities of PCI (1–3) must be thoroughly understood, respected and considered when developing or using motion-based technologies with these populations. For example, impairments in attention, concentration, visuospatial abilities, working memory, and cognitive processing speed can impact the way PCI experience and interact with motion-based technology (1–3). These cognitive changes can interfere with the speed at which people learn, how much training they need, and how they perform against standard benchmarks. However, PCI can learn to use motion-based technology with the right prompting and support (7). Thus, it is ideal to design or use motion-based technologies such that the cognitive needs of PCI are accommodated in order to increase accessibility and usability of the device and software. From the literature, cognitive considerations for PCI relative to healthy controls include: loss of executive function, and specifically working memory; leveraging remaining cognitive abilities (e.g., procedural and working memory); utilizing prompts which have been shown effective with PCI; accounting for processing speed differences; and accommodating impairments in attention and concentration (14, 17). We now explore these considerations in terms of relevant design and use implications.

#### Choose a goal or task that is clear, engaging and achievable

Executive function is the cognitive process that enables people to plan, focus attention, remember, and manage multiple tasks. As executive function is commonly impaired in PCI, motion-based technology systems must be designed to ensure that demands on executive functioning are not excessive (44). For example, impairments in executive function affect one's ability to follow along or complete several tasks simultaneously, and thus, motion-based technologies designed for use with PCI should feature a clear, understandable and achievable goal, with the task (e.g., throwing a bowling ball down a virtual bowling alley) directly related to the overall goal (e.g., knocking down bowling pins) (35). Furthermore, activities presented on motion-based technology should be straight-forward and familiar, such as digital versions of existing games (e.g., bowling) or activities that reflect the person's hobbies and interests (29, 30, 39). Ideally, the complexity of the activity and the abilities of the population should remain in balance (i.e., not too easy, not too hard) (26, 29, 30, 35, 39) while also remaining engaging, challenging and stimulating enough to sustain attention (27, 35, 39). This requires a good understanding of the activity and what demands it places on different aspects of cognitive function.

#### Maximize retained skills and limit involvement of impaired skills

Motion-based technology systems must be responsive to the cognitive needs of PCI by maximizing involvement of spared abilities (e.g., procedural memory) and minimizing involvement of impaired abilities (e.g., working memory) (26, 35). For instance, to fully harness the potential of spared procedural and errorless learning capabilities in PCI (1), it is recommended that motion-based technology system designs promote and support errorless learning (i.e., guiding the person toward the right answer rather than relying on trial and error) and procedural learning (e.g., frequently practicing the gestures) (26, 31, 36, 37). For example, Dove and Astell (45) created a methodology tailored to PCI, helping caregivers teach PCI to play a digital bowling game on motion-based technology by breaking down the entire movement sequence (i.e., grabbing a bowling ball and throwing it down the lane) into procedural steps. This tailored approach could easily be integrated into motion-technology systems by using in-task prompts to support the user through the activity, especially given that verbal prompts and cues are commonly used to teach PCI to use motion-based technologies (28, 30–32, 35–37, 40, 41, 43). However, it is imperative to avoid incorporating too many or too few steps required to achieve the task, as the activity may become discouraging or unappealing to PCI (26).

#### Ensure that instructions are appropriate and understandable

Working memory, a component of executive functioning, is the part of short-term memory concerned with immediate conscious perceptual and language processing. As working memory is significantly impaired in populations with dementia and often an area of concern in MCI (46), motion-based technology systems must be designed to ensure that demands on working memory are low (i.e., minimize amount of information and duration that it has to be kept in mind). As such, motion-based technology systems designed for PCI should offer instructions that use common, clear and concise language (e.g., avoid technical language, irrelevant information or excessive use of text) (26, 35, 37, 39). Instructions should clearly explain the task objective and the steps required to meet this goal using a combination of visual (i.e., graphical) and written instructions (26, 27, 35, 39, 40). Additionally, instructions in the form of on-screen demonstrations, text and audio-visual cues can be used to teach PCI to use motion-based technology (25, 27, 28, 35, 39). For example, the software should offer on-screen gesture demonstrations at the beginning of an activity in addition to cues during the activity, rather than expecting the person to recall the gestures (26, 35). While this may be considered repetitive for healthy populations, the repeated demonstrations are important for PCI. However, including too much information or too many methods of instruction at once may become overwhelming or distracting for PCI, and is therefore cautioned against (26, 35, 37, 39).

#### Ensure that prompts are effective and enabling

Prompts (or reminders) are frequently used to coach PCI through tasks (e.g., making tea, playing bowling on Xbox Kinect) and have proven successful in doing so (7, 45). Thus, it is suggested that prompts be incorporated into motion-based technology system designs to support PCI to complete tasks with motion-based technology (28, 30–32, 35–37, 40, 41, 43). Moreover, system designs must ensure that in-task prompts enable PCI to actively participate with the highest degree of independence rather than simply completing the task for them (39). For example, given the immersive nature of motion-based technology, system designs must ensure that prompts are not given excessively (i.e., give the person a chance to attempt the task on their own first) or at inappropriate times (e.g., when the person is in the middle of performing a task) (13, 26, 35). It is recommended that different prompting methods and timings are created and trialed with PCI to evaluate their effectiveness.

#### Avoid timed responses and complex interactions

To account for reduced cognitive processing speed in PCI, motion-based technology software and interventions should avoid using time limits, to allow players to interpret information on the screen and elicit a response at their own pace (26, 35). Additionally, it is advised that motion-based technologies designed for PCI avoid tasks that require quick and/or complex cognitive responses (26, 30, 35). Avoiding tasks or objectives that involve multiple cognitive domains at once (e.g., motor coordination, visual attention, speed, working memory) is also suggested (26, 35, 39), in addition to minimizing methods of interaction (e.g., not having too many buttons, actions, or options to choose from) (26, 37, 39).

#### Gain and sustain the attention of the user

To accommodate impairments in attention and concentration in PCI, special attention should be paid to creating scenes, activities and prompts that can capture and sustain attention. For example, after a period of inactivity, it might be helpful if the system were to prompt the player using audio-visual effects in order to draw them back into the gaming interaction (39). While designs must be engaging and stimulating enough to capture and sustain attention (35, 39), it is equally pertinent to avoid creating games and interfaces that look overly complicated or cluttered (e.g., too many icons on the screen or complex backgrounds) as this may serve as a distraction or confuse people as to which item on the screen they should focus on Benveniste et al. (26), González-Palau et al. (32), Konstantinidis et al. (35) and Siriaraya and Ang (39). Furthermore, it is also important to avoid the use of distracting in-game features such as overpowering background music or disruptive pop-up messages (26, 35, 39).

#### Reduce or eliminate the possibility of failure

It has been well-established that making games “failure-free” (i.e., making failure either impossible or highly improbable) is extremely important when designing activities for PCI, as failure can cause discouragement, frustration and may deter the person from participating in the activity again (26, 31, 35, 37). This principle is naturally extended to any technological system that is designed for PCI. If motion-based technology systems have the possibility of error or “failure,” the feedback provided to the user as a result of the error should be mitigated, and encouragement should be provided to promote continued participation. An example of this design principle is found in the MINWii system, a motion-based music therapy game for people living with dementia (26, 37). Any wrong note that is chosen by participants is played at a much lower volume than the rest of the notes, rather than emphasizing the error. Furthermore, it is suggested that motion-based technology avoid using scoring metrics such as points, wins/losses and pass/fail decisions for the interaction component of the system (26). The focus of the participant interface of motion-based technologies should be to build confidence and empower PCI, while scoring or evaluations should take place behind the scenes (26, 35). In some cases, minimal in-activity scoring is recommended in group activities involving PCI—such as Xbox Kinect bowling groups—as scoring can evoke mild yet friendly competition, which enhances the leisure experience (7, 35, 45). The relevance and appropriateness of including such feedback will vary by activity.

### Physical domain considerations

In addition to the cognitive considerations when working with people who have MCI or dementia, it is also necessary to consider the physical effects as well as those from aging, as age is the main risk factor for developing dementia or MCI (2, 3). For example, the presence of dementia or MCI can result in deficits in physical functioning such as motor control, gait, fine motor coordination, speed, and balance (34, 35, 38). Specifically, the literature pertaining to motion-based technology considers mobility aids, reduced motor control, speed and reaction time, an inability to process complex or repetitive motions, and reduced visual and auditory capabilities in populations of PCI. While many studies recommend the use of trained therapists to facilitate motion-based technology interventions with PCI (25, 27, 33, 34, 36, 38, 41, 42), the impact of the physical changes associated with PCI on the ability to interact with the technology can be accommodated and integrated into the technology design for this population. We now consider the effects of these factors on the design and use of motion-based technologies.

#### Accommodate mobility aids

When considering motion-based technologies for use with PCI, it is important to ensure that all activities offered are physically accessible and allow players with a wide range of physical abilities to participate (7, 26, 35, 45). Several studies exploring the use of motion-based technology for PCI highlighted the need for systems that accommodate both standing and seated play (26, 35, 39, 45) in addition to successfully accommodating mobility devices such as walkers and wheelchairs into system interactions (29, 35, 39, 45). For example, Dove and Astell (45) found that the Xbox 360 Kinect used in their study was unable to accommodate seated participants or those who used assistive devices. This resulted in participants with mobility impairments relying on caregivers to physically support them from behind while they stood and interacted with the system. In these cases, the technology is not able to meet the physical needs of the person, resulting in instances where caregivers need to be more involved in the interaction than the person it was intended for. However, more recently, Dove and Astell (47) used the newer Xbox One Kinect to run group activities for PCI and found that the technology was more accessible for people who require mobility devices or seated play than the Xbox 360 Kinect, allowing participants with a wide range of abilities to engage with the technology independently.

#### Account for inaccurate or imprecise motor control

Motion-based technologies applied to PCI must accommodate a greater “window of accuracy,” offering a wider range of movement allowance rather than relying on precise motions for interaction (26, 29, 35, 39). For instance, following a study using the Xbox Kinect, Dove and Astell (45) highlighted the need for motion-based technology software to accommodate restrictions in range of motion, as some participants with dementia found it difficult to raise their arm high enough to activate the system. The result is that PCI could not use the system, potentially missing out on the benefits of the intervention. By creating systems that accommodate the physical limitations and reduced motor control of PCI, motion-based technology will become more accessible and usable for these populations, allowing them to engage with the highest degree of independence.

#### Ensure that the physical component is age-appropriate

Due to impairments in speed and reaction time related to age, dementia or MCI, motion-based technologies for PCI should minimize the use of time-sensitive features requiring fast motor responses (26, 35) and complex, repetitive or extensive movements (e.g., involving several different limbs) that may result in fatigue or overexertion (26, 35, 39). Again, this can be accomplished by leveraging residual procedural memory (31, 36, 37), by breaking complex tasks into sequences and providing an appropriate amount of time to complete each task step. Furthermore, to keep the demand on the motor system low, it is recommended that gestures incorporated into motion-based technology for PCI are simple, familiar, and well-defined rather than supporting a variety of gesture options (35). An example of a motion-based technology that is age-appropriate is the Xbox One Kinect bowling game, which was used in Dove and Astell (45) to run group activities for people with dementia who attend adult day programs. The entire interaction with the software only relies on six simple, well-defined gestures which include; raising an arm above the head, extending the arm out to the side, closing the hand to grab the ball, extending the arm backwards, swinging the arm forward, and opening the hand to throw the ball. None of these steps are time-sensitive, allowing participants to interact with the technology at their own pace.

#### Create interfaces and interactions that are intuitive and realistic

Lack of opportunities to participate in meaningful leisure activities is a common challenge experienced by PCI (6). Additionally, it can be harder to achieve sustainable engagement in PCI due to impairments in attention (6). Simple, immersive, user-friendly, intuitive, and naturalistic interactions and interfaces have been shown to facilitate learning, flow of play and enjoyment in PCI (26, 35). For example, in several studies, the use of a “hands-free” motion-based technology (e.g., Xbox Kinect) is recommended for use with PCI due to the accessibility and realistic nature of the technology (26, 35, 39–41). Furthermore, due to decreased fine motor skills and manual dexterity, it is advised that the use of any kind of hand-held device (e.g., a game controller) is avoided with motion-based technology for PCI (26, 35, 39–41). While motion-based technologies that operate through hand-held controllers (e.g., Nintendo Wii) have been reported as usable and enjoyable by PCI (26, 29, 30, 33, 34, 41), these types of devices create interactions that are less intuitive and harder to learn for PCI. Having to recall the functions of multiple buttons and coordinating the pressing of buttons with the execution of physical motions places additional demands on their already restricted cognitive resources. For example, in a study conducted by Tobiasson et al. (41), the hand-held controller used to interact with the motion-based technology had to be modified by covering most of the buttons with a thermoplastic splint to make the controller easier to hold and to prevent PCI from unintentionally pressing the wrong buttons.

#### Accommodate variations in participant capabilities

As PCI represent a very diverse group, it is advised that systems developed using motion-based technology be adaptable and adjustable to suit a wide range of cognitive and physical abilities (26, 32, 35, 40). For example, the system could use activity recognition and player profiles to tailor the experience for each individual (17). Adaptable motion-based technology designs were similarly recommended by Nansen et al. (48), who explored the use of Microsoft Kinect for older adults. However, an adaptive approach may not always be feasible (e.g., in a group setting) or necessary (e.g., when using the technology for leisure activities rather than cognitive or physical exercises). Additionally, if an adaptable approach is taken, it has been suggested that the system be designed to account for the current and future needs of the person it is adapting to Bamidis et al. (25), Billis et al. (27), González-Palau et al. (32) and Leahey and Singleton (35). That is, as a PCI's cognitive and/or physical abilities continue to deteriorate over time, the system could accommodate these changes and support the person by adjusting the level of difficulty, interaction and interfaces, aesthetic design, and method of instruction and prompts.

#### Include visual and auditory accommodations

In addition to general age-related physical considerations (e.g., decrements in fine motor skills), age-related visual and auditory impairments (e.g., vision loss, hearing loss) must also be considered when using motion-based technologies with PCI (2, 3). To accommodate visual and auditory changes, interfaces should feature simple yet engaging scenes with backgrounds, text and graphics that are well-defined, highly contrasted, and bright or pastel-colored (26, 35, 39). Furthermore, fonts, icons, graphics, and symbols must be large enough to be easily seen (26, 30, 35, 37) in addition to avoiding the use of small or fast-moving objects. Excessive features, or “eye candy” should be kept to a minimum in order to create in-game scenes that are engaging but not distracting or cluttered (26, 35, 39). The interface should be flexible for people with visual or hearing impairments by allowing users to increase the size of objects or text, in addition to changing the volume or pitch of auditory content (e.g., background music, voice commands) (26, 35, 39). Furthermore, prompts should be available in a variety of forms (e.g., text or sounds) to accommodate the individual auditory and visual needs of PCI.

### Social domain considerations

In many cases, motion-based technology interventions must be responsive to the social needs of PCI, which includes addressing people's needs for social interaction and active participation in their environment. For example, PCI commonly experience impairments in communication, which affect their ability to initiate and participate in social interactions or conversations with others (49). These impairments will impact interactions with technologies as well. However, despite these challenges, PCI also have an undiminished need and desire for these social contacts. It is ideal to develop technologies that address this social need while compensating for the unique social challenges experienced by PCI. Motion-based technologies are ideally suited for this application, supporting systems that, for example, can be used in groups (33, 35, 39, 41, 43). An example of this can be identified in recent work by Mueller et al. (50), whose findings revealed that embedding a social component in motion-based gaming interventions can increase motivation and engagement. Relevant social considerations for PCI include a focus on group activities, ensuring timely and constructive interactions with the system, and reducing the likelihood of failure during system interactions. We now consider these social factors in the context of design and use implications for motion-based technologies.

#### Tailor the activity to the person's interests

People have different interests and preferences about how they wish to spend their time, and unsurprisingly, PCI are no exception (51). Therefore, to facilitate engagement in motion-based technology interventions, especially if utilized in a leisure context, it is important to choose activities that are relevant and meaningful to PCI (i.e., playing music, playing a favorite sport) (26, 29, 30, 35, 39). Activities offered to PCI should aim to be pleasant and engaging, while promoting independence and mastery, self-confidence and autonomy (30, 33, 39, 41). To fully understand what activities are meaningful and relevant to PCI, their input should be included throughout the design process (i.e., participatory design) and considered when selecting an activity (e.g., asking the person what activities they enjoy) (12).

#### Design for an audience

Presenting motion-based technology as a group activity can provide an array of social benefits for PCI, which positively contributes to the leisure experience (7). For example, in several studies exploring the use of motion-based technology for PCI, presenting the technology as a group activity was found to promote social interaction, maintain social skills, and reduce social barriers (7, 39). In addition, using motion-based technology in a group setting can encourage friendly competition and intergenerational connections for PCI (7, 35, 45). This suggests that motion-based technology systems should be designed or used to promote and support social interaction either between players, or between players and caregivers (26, 30, 35, 39, 41). This could be achieved by creating systems that simultaneously allow multiple people to participate or allow others to engage peripherally by observing the activity (45).

#### Ensure system interactions are timely, constructive and positive

Interactions between motion-based systems and PCI can occur in many forms including audiovisual performance feedback, prompting, and cues. For example, feedback (i.e., a response related to a person's performance of a task) is used in digital gaming to promote learning and player motivation. It is crucial to provide the right type of feedback at the right time when interacting with PCI. For example, feedback that occurs immediately after an event is encouraged over delayed feedback (35, 39). Indeed, feedback or prompts provided to PCI from motion-based systems can be confusing or ignored if inappropriately timed (13). Feedback should be positive, encouraging and communicated through features such as music playing, people cheering, motivational messages, or encouraging graphics (26, 28, 30, 35, 39, 41). More importantly, the use of negative feedback (e.g., an avatar shaking its head) should be avoided as it may be discouraging for PCI, which may dissuade long-term engagement or result in abandonment (26, 28, 35, 39). Multimodal feedback, such as audio-visual feedback (i.e., feedback related to both hearing and sight) is encouraged (26, 35, 39). For example, when designing or using motion-based technology for PCI, feedback using technologic effectors (e.g., visual effects, sounds) can be used to direct the attention of a PCI to a specific object, area or task (39). Additionally, in the case of interactive systems with visual displays, feedback may also be supplied in the form of text, such as having the words “good job!” appear on the screen upon completion of a step or task (26). Timely, constructive and positive feedback and prompting is critical in motion-based technology systems because PCI are interacting with the system without any physical connection.

## Discussion

There is an urgent and growing need for interventions to support rising numbers of PCI. In the absence of pharmacological solutions, the potential of technology to offer interventions for cognitive, physical and social challenges of later life is increasingly being recognized. Here we offer a set of guidelines for improving, maximizing and speeding up the utilization and implementation of motion-based technologies for PCI. Additionally, our guidelines seek to increase the usability of current motion-based technology systems and software for PCI. In particular, we present our findings within three domains of consideration: cognitive, physical and social. Within each domain, we consider specific themes that have emerged in the literature that are unique to PCI relative to healthy individuals. These evidence-based guidelines respond to a lacuna in the literature regarding the specific design considerations relevant to systems using motion-based technologies with PCI (7).

Our findings shape how researchers and developers introduce, teach and use motion-based technologies with PCI. Specifically, these guidelines seek to inform developers of motion-based technologies and researchers using these technologies, resulting in improved data collection and participant engagement. From these guidelines, it can be understood that there are many considerations specific to PCI that may affect their ability to interact with motion-based technologies. While these exploratory guidelines are not exhaustive due to the limited number of articles identified in the literature (*n* = 19), they serve to pave the way for future investigations. For example, the varying amount of detail provided in each article and the variance in sample size makes it hard to synthesize and compare this body of literature, especially given that this area of research is relatively new in nature. However, in the absence of pharmacological or other interventions for PCI, this work is significant given that it has the potential to open up a wide range of interventions to support cognitive, physical and social/communication difficulties within these populations.

Notably, the design themes are not exclusive, meaning that improving a system in one domain for a specific consideration may reduce the system's efficacy in another domain. Furthermore, the themes also involve a balance between accommodating a deficiency and overloading another domain. An example of this is providing audiovisual feedback to gain and sustain the attention of the user. Too much feedback may cause confusion or distraction for PCI, as too many attention-grabbing aspects can distract the person from the primary goal or objective. However, the guidelines presented here highlight several potential design opportunities that can address these changes in order to increase the accessibility and usability of motion-based technology systems for PCI.

As previously stated in the literature (7), motion-based technology can provide cognitive stimulation, promote physical activity participation and create meaningful leisure activities for PCI; all of which can contribute to their well-being and an improved quality of life. Furthermore, PCI can learn to interact with motion-based technologies and enjoy doing so (7, 45). This suggests that motion-based technology can be used as a fun tool to improve well-being for PCI, and also serve as an effective tool for unobtrusive research. Thus, using appropriate system design to support PCI to use motion-based technology will increase the benefit of this type of technology even more so.

While these guidelines present many potential avenues for progress, this area of research still requires further investigation. For instance, to suitably integrate the needs of PCI in motion-based technology systems, technology designers must consider the end-users needs throughout the entire process (i.e., conception to implementation), which also includes involving PCI in future research regarding motion-based technology design (7). By keeping the target audience in mind during all phases of the design process, the likelihood that the system will support, benefit and meet the needs of these groups is substantially improved; thereby increasing the potential of the motion-based technology for PCI (13).

## Conclusions

Motion-based technology has great potential for PCI, but is currently underexplored and underutilized. To advance this we propose a set of evidence-based guidelines. To improve the user-friendliness and practicality of this type of technology for PCI, three key areas—cognitive, physical and social—must be integrated into aspects of motion-based technologies (e.g., meeting the social needs of PCI by designing games that promote social interaction and meaningful engagement). Hopefully, this will further increase the ability of motion-based technology to support an improved life for PCI.

## Author contributions

All authors listed have made a substantial, direct and intellectual contribution to the work, and approved it for publication.

### Conflict of interest statement

The authors declare that the research was conducted in the absence of any commercial or financial relationships that could be construed as a potential conflict of interest.
